# Egg Speckling Patterns Do Not Advertise Offspring Quality or Influence Male Provisioning in Great Tits

**DOI:** 10.1371/journal.pone.0040211

**Published:** 2012-07-17

**Authors:** Mary Caswell Stoddard, Annette L. Fayet, Rebecca M. Kilner, Camilla A. Hinde

**Affiliations:** 1 Department of Zoology, University of Cambridge, Cambridge, United Kingdom; 2 Oxford Navigation Group, Department of Zoology, University of Oxford, Oxford, United Kingdom; 3 Edward Grey Institute, Department of Zoology, University of Oxford, Oxford, United Kingdom; University of Melbourne, Australia

## Abstract

Many passerine birds lay white eggs with reddish brown speckles produced by protoporphyrin pigment. However, the function of these spots is contested. Recently, the sexually selected eggshell coloration (SSEC) hypothesis proposed that eggshell color is a sexually selected signal through which a female advertises her quality (and hence the potential quality of her future young) to her male partner, thereby encouraging him to contribute more to breeding attempts. We performed a test of the SSEC hypothesis in a common passerine, the great tit *Parus major*. We used a double cross-fostering design to determine whether males change their provisioning behavior based on eggshell patterns they observe at the nest. We also tested the assumption that egg patterning reflects female and/or offspring quality. Because birds differ from humans in their color and pattern perception, we used digital photography and models of bird vision to quantify egg patterns objectively. Neither male provisioning nor chick growth was related to the pattern of eggs males observed during incubation. Although heavy females laid paler, less speckled eggs, these eggs did not produce chicks that grew faster. Therefore, we conclude that the SSEC hypothesis is an unlikely explanation for the evolution of egg speckling in great tits.

## Introduction

**Figure 1:**
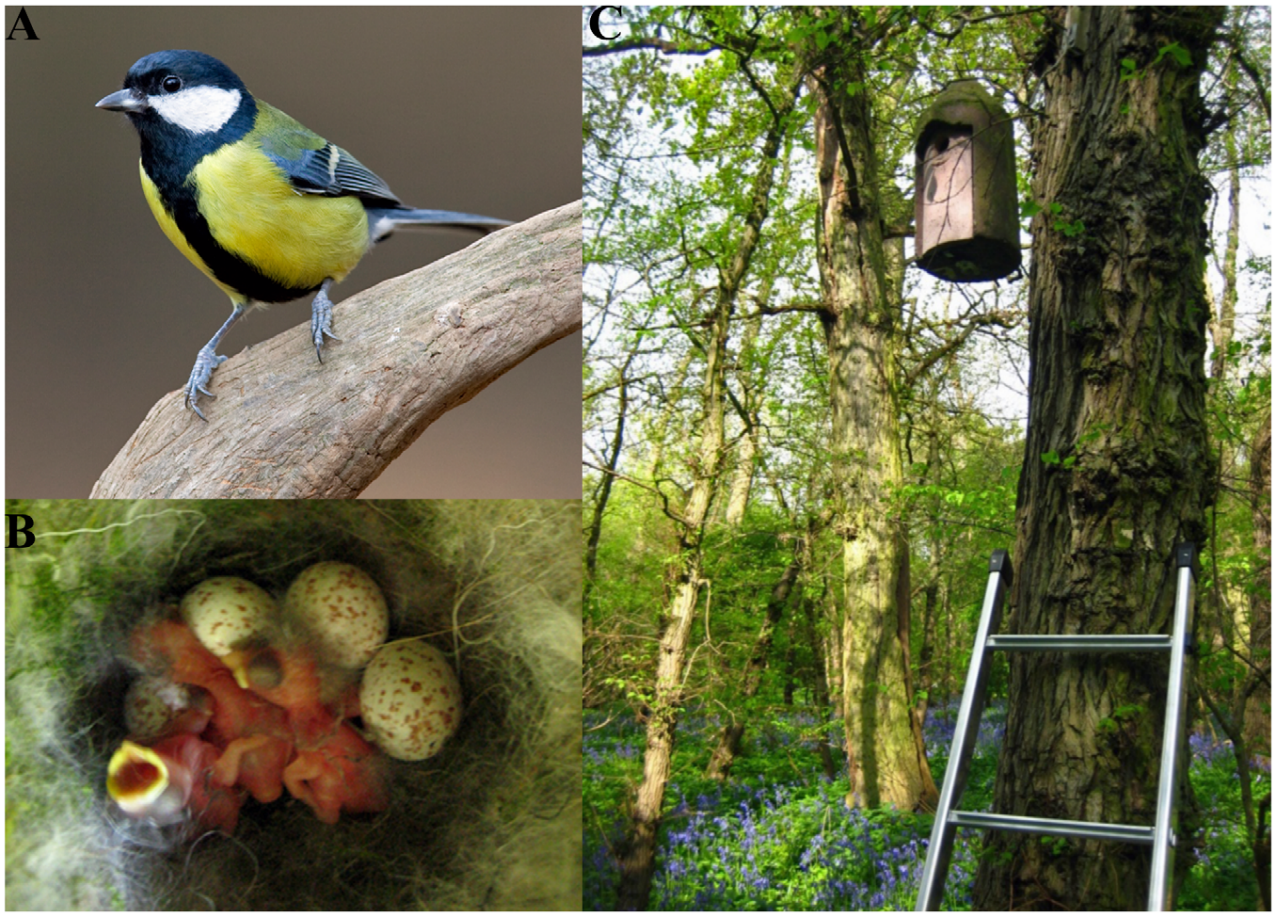
Madingley Wood in Cambridgeshire (UK) is a popular nesting site for great tits (*Parus major*). **(A)** A male great tit. **(B)** A nest (day  = 0) containing newly hatched chicks as well as several eggs that have not yet hatched**. (C)** A woodcrete nestbox in Madingley Wood. Photo credits: D. Kjaer (A) and M. C. Stoddard (B-C).

The evolution of speckled eggs has occurred in many avian lineages, a phenomenon often attributed to selection for eggs that are camouflaged and cryptic [1], [2]. While this might explain why many open-nesting species lay patterned eggs, it does little to clarify why hole-nesting passerine species in multiple lineages exhibit egg speckling [1]. The great tit *Parus major* epitomizes this conundrum. Great tits lay white eggs with reddish-brown speckles produced by protoporphyrin pigment [3] (Figure 1). Because great tits nest in holes or cavities, and because females typically conceal their eggs during the laying period (when no incubation occurs) by covering them with nest material, it is unlikely that speckling helps to provide camouflage. Furthermore, the great tit is not a host of a brood parasite [4], so egg speckling is not explained by the coevolutionary arms race between brood parasites and their hosts. What purpose, then, do these pigmentation patterns serve?

Several signaling and structural hypotheses have been proposed [1], [5], [6], [7], [8]. Among these, the sexually selected eggshell coloration (SSEC) hypothesis has spawned a flurry of research interest, with dozens of papers published on this idea since its initial conception by Moreno and Osorno [9]. The SSEC hypothesis posits that eggshell coloration is a sexually selected signal through which a female advertises her quality, and hence the quality of her nestlings, to her male partner, thereby persuading him to contribute more to the breeding attempt after mating [9]. In birds, egg coloration principally stems from two pigments: biliverdin, an antioxidant bile pigment responsible for blue-green coloration, and protoporphyrin, a pro-oxidant pigment responsible for red-brown coloration and patterning (maculation) [1]. Both pigments are products of the biosynthesis of heme, an iron-containing component which plays a vital role in oxygen transport and storage in the blood stream of vertebrates [10]. In its original formulation, the SSEC hypothesis proposed that a female might broadcast her antioxidant capacity by depositing generous amounts of blue-green biliverdin pigment on her eggs. Tests of the SSEC hypothesis for blue-green pigmentation have been equivocal: some studies have demonstrated an association between the intensity of blue-green egg coloration and male provisioning behavior [11], [12], [13], [14], [15], [16], while others have found no such relationship [17], [18], [19], [20]. The studies listed here include both correlational and experimental studies. For detailed reviews, see [7], [21].

Moreno and Osorno [9] left open the possibility that the SSEC hypothesis might also apply to red-brown protoporphyrin pigmentation. This extension of the SSEC hypothesis initially received little attention; however, several recent studies have revisited the idea that protoporphyrin-produced spotting or speckling may too be a sexual signal. So far, these studies have been largely inconclusive. One obstacle is that protoporphyrin production and deposition are still poorly understood [22], making it difficult to predict whether increased protoporphyrin deposition on eggshells should reflect poor or good female condition. Unlike antioxidant biliverdin, protoporphyrin is a pro-oxidant that may induce oxidative stress in females [23]. Heavily speckled eggs may indicate a female’s inability to remove harmful protoporphyrin molecules from the system, thus advertising poor health. Alternatively, heavily speckled eggs may signal a female’s ability to sustain high levels of stress or effectively remove the stressors, thus indicating good health [9]. In the first direct test of the SSEC hypothesis for egg speckling, Martínez-de la Puente et al. [24] proposed that protoporphyrin speckling was an indicator of female stress and suggested that males should invest less in offspring hatching from heavily speckled eggs. Female blue tits *Cyanistes caeruleus* in poorer body condition laid spottier eggs and were paired with males in poorer condition; however, the authors did not test whether males altered their provisioning behavior in response to egg patterning [24]. More recently, Walters and Getty [25] found in house wrens *Troglodytes aedon* that brighter, less speckled eggs were heavier and produced chicks that were better fed by mothers but not fathers, although this experiment did not control for possible confounding effects of nestling quality (see below).

**Figure 2:**
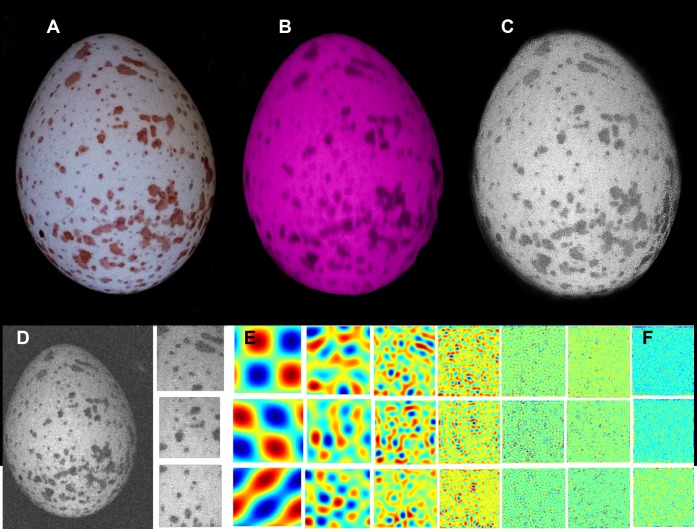
Great tit eggs were photographed in the wild using a UV-sensitive camera, producing images in the (A) human-visible spectrum and (B) ultraviolet spectrum. Images were converted to correspond to a blue tit’s (*Cyanistes caeruleus*) double cones **(C)**. Egg patterns were measured using a digital image analysis technique **(D)** based on the fast Fourier transform, in which information about egg speckling is captured at different spatial frequencies. The original image is broken down into seven new images, each containing information at a different spatial scale. Low spatial frequencies **(E)** capture information about the relative contribution of large markings and high spatial frequencies **(F)** capture information about the relative contribution of small markings (see Stoddard and Stevens 2010 for details). Egg photograph by M. C. Stoddard.

In contrast to studies suggesting that increased protoporphyrin speckling may signal poor female quality, Sanz and García-Navas [26] found that high-quality female blue tits tended to lay eggs with darker speckles and that males (but not females) had higher provisioning rates at nests where eggs had a high degree of ‘pattern spread.’ It is not clear, however, whether males increased visit rate in response to egg pattern, chick quality, or because they themselves were high-quality individuals. Consistent with these results are those from a study of captive house sparrows *Passer domesticus*. In this study, López-de-Hierro and De Neve [27] also reported a positive association between the extent of protoporphyrin speckling and aspects of female quality, such as clutch size. However, spots were also darker at the end of the breeding season, presumably when female quality had declined, so if egg speckling advertises female condition in house sparrows, it does so in a complex way.

**Table 1:** General Linear Model showing the amount of prey delivered by males was not related to the speckling on the eggs his partner laid or the eggs that he saw during incubation (N = 29 nests).

*Dependent – Amount of prey* *delivered by male*	B	df	F	95% L conf. int.	95% U conf. int.	P
Intercept	0.54	1/27	11.20	−0.29	0.36	0.99
Brood size (experimental)	0.54	1/27	11.20	0.21	0.86	**0.002**
Amount of prey delivered by female	0.30	1/26	2.32	−0.10	0.70	0.14
Clutch size	0.18	1/26	1.10	−0.17	0.52	0.30
Egg speckling (PC1) female laid	−0.12	1/26	0.61	−0.44	0.20	0.44
Lay date	−0.05	1/26	0.08	−0.41	0.32	0.79
Egg speckling (PC1) female incubated	−0.04	1/26	0.06	−0.38	0.30	0.81
Average egg mass	−0.02	1/26	0.01	−0.34	0.31	0.92

**Table 2:** DAY 3. Linear Mixed-effects Model showing no effect of egg speckling on chick mass on day 3.

*Dependent – Chick mass on day 3*	B	df	F	95% L conf. int.	95% U conf. int.	P
Intercept	−0.01	58.71	0.02	−0.19	0.17	0.90
Lay date	−0.19	48.05	3.76	−0.39	0.01	0.06
Brood size (experimental)	0.06	62.67	0.52	−0.11	0.24	0.48
Original egg speckling (PC1)	0.07	58.81	−.49	−0.12	0.26	0.49
Chick sex male	0	–	–	–	-	-
female	0.06	211.98	0.21	−0.19	0.30	0.65
Average egg mass	0.03	58.33	0.11	−0.15	0.21	0.74
Egg speckling (PC1) foster parent laid	0.03	59.91	0.10	−0.15	0.20	0.75
Clutch size	−0.02	59.82	0.06	−0.21	0.17	0.81
Clutch size - Foster female	−0.01	57.43	0.03	−0.16	0.13	0.85
Egg speckling (PC1) foster parent incubated	−0.01	62.67	0.01	−0.21	0.19	0.93

Original nest nested within foster nest was included as a random factor (estimate 0.28, +/-SE 0.09). N = 287 chicks and 47 nests.

**Table 3:** DAY 7. Linear Mixed-effects Model showing no effect of egg speckling on chick mass on day 7.

*Dependent – Chick mass on day 7*	B	df	F	95% L conf. int.	95% U conf. int.	P
Intercept	0.04	56.72	0.17	−0.14	0.21	0.68
Lay date	−0.27	49.58	7.90	−0.46	−0.08	**0.007**
Clutch size	−0.19	58.34	4.44	−0.36	−0.01	**0.039**
Average egg mass	0.16	57.09	3.46	−0.1	0.32	0.07
Egg speckling (PC1) foster parent laid	−0.06	59.63	0.51	−0.23	0.11	0.48
Original egg speckling (PC1)	−0.06	57.44	0.48	−0.24	0.12	0.49
Egg speckling (PC1) foster parent incubated	0.06	59.59	0.48	−0.12	0.25	0.49
Clutch size - Foster female	0.05	56.10	0.45	−0.09	0.19	0.51
Chick sex male	0	–	–	–	–	–
female	−0.05	205.13	0.21	−0.28	0.17	0.64
Brood size (experimental)	0.02	68.63	0.04	−0.16	0.20	0.85

Original nest nested within foster nest was included as a random factor (estimate 0.27, +/-SE 0.08). N = 266 chicks and 47 nests.

**Table 4:** DAY 15. Linear Mixed-effects Model showing no effect of egg speckling on chick mass on day 15.

*Dependent – Chick mass on day 15*	B	df	F	95% L conf. int.	95% U conf. int.	P
Intercept	0.34	53.65	0.25	0.15	0.53	0.70
Chick sex male	0	–	–			–
female	−0.59	180.91	41.56	−0.77	−0.41	**<0.001**
Lay date	−0.38	49.71	15.55	−0.57	−0.19	**<0.001**
Average egg mass	0.25	54.13	8.68	0.08	0.43	**0.005**
Clutch size	−0.25	53.90	7.59	−0.42	−0.07	**0.008**
Brood size (experimental)	−0.14	54.48	2.35	−0.31	0.03	0.13
Egg speckling (PC1) foster parent incubated	0.11	53.91	1.58	−0.07	0.29	0.22
Original egg speckling (PC1)	−0.07	52.08	0.52	−0.26	0.12	0.47
Clutch size - Foster female	−0.05	48.82	0.45	−0.19	0.09	0.51
Egg speckling (PC1) foster parent laid	−0.05	54.10	0.38	−0.22	0.12	0.54

Original nest nested within foster nest was included as a random factor (estimate 0.30, +/−SE 0.08). N = 236 chicks and 47 nests.

Why have recent empirical tests generated such mixed results? Evaluation of the SSEC hypothesis has faltered for three main reasons. For more extensive reviews, see [7], [8]. The first problem is that most studies have been correlative, rendering the reported relationships “intriguing but impotent” [7]. Great tit males and females are known to mate assortatively [28], so egg phenotype need not be causally related to male provisioning rates but rather the consequence of high-quality males and females pairing up in the first place. A handful of tests of the SSEC hypothesis for biliverdin pigmentation have employed cross-fostering approaches to decouple the effects of egg color and maternal quality [13], [17] – and in the case protoporphyrin see [25] – but even these approaches do not go far enough because they fail to control for the potentially confounding effect of nestling quality on paternal investment [21]. In these studies, it is impossible to determine whether parental feeding rates are associated with egg color or with other correlates of chick quality, such as gape color, nestling size, and begging intensity [29].

**Figure 3:**
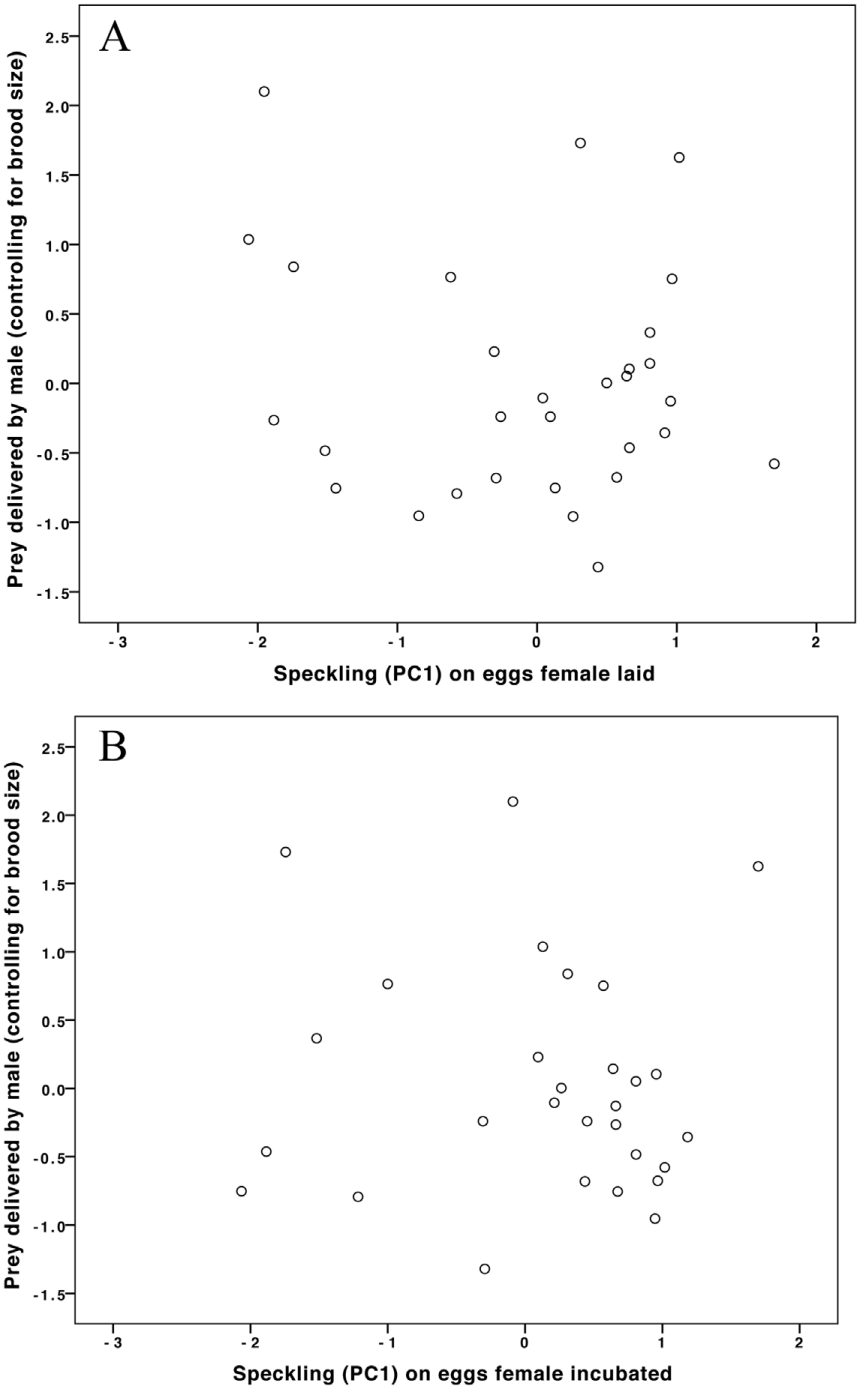
The amount of prey delivered by males was not related to egg speckling on the eggs (A) that his partner laid or (B) that he saw (his partner incubated). N = 29 nests. Brood size is controlled for by using the residuals of prey delivered by males over brood size on the y-axis.

To determine clearly whether egg patterning is related to female quality, it is essential to perform experimental manipulations in place of correlative studies. Moreover, to decouple male provisioning and egg coloration without the confounding effects of nestling characteristics, eggs should be swapped twice: once after they are laid (to break the correlation between egg appearance and maternal quality) and again after they hatch (to break the correlation between egg appearance and nestling quality) [21]. Two studies on blue-green biliverdin pigmentation have employed such an approach [15], [16]; in each experiment, natural clutches were replaced with artificial eggs and unrelated chicks were subsequently added back into nests at the end of the incubation period. The one drawback to these studies is that they used artificial eggs, which can introduce experimental artifacts, *i.e.*, model eggs rarely match the true color and pattern of natural eggs and can be problematic in experimental studies [5], [8], [30].

The second shortcoming of studies investigating the SSEC hypothesis is their reliance on human assessments of color and pattern or on reflectance spectra, both of which fail to account for bird vision. Consequently, such studies have not evaluated egg appearance as it appears to the relevant receiver: in this case, the male bird. This has been particularly true for egg patterning, as opposed to egg coloration, because objective measurements of marking size and marking distribution have been historically difficult to obtain. As a result, pattern measures in most previous studies have been derived from human-produced rankings. Several studies related to egg rejection and brood parasitism have successfully employed models of avian vision for color [31], [32], [33] and pattern [34], [35], but tests of the SSEC hypothesis have been slow to incorporate visual modeling – see [36] for an exception.

**Figure 4:**
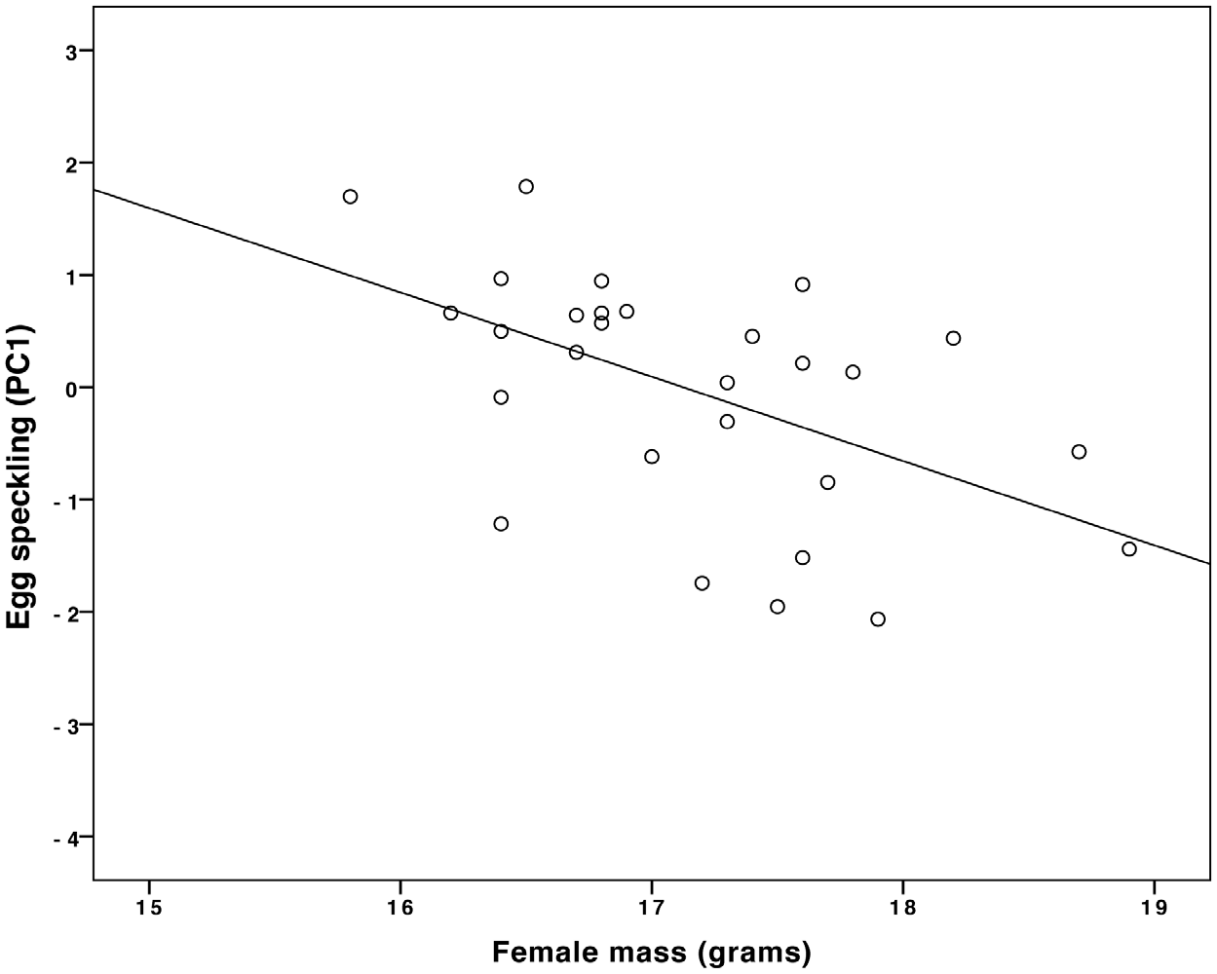
Heavier females laid less speckled eggs (PC1). N = 28 nests (see text for statistics).

**Figure 5:**
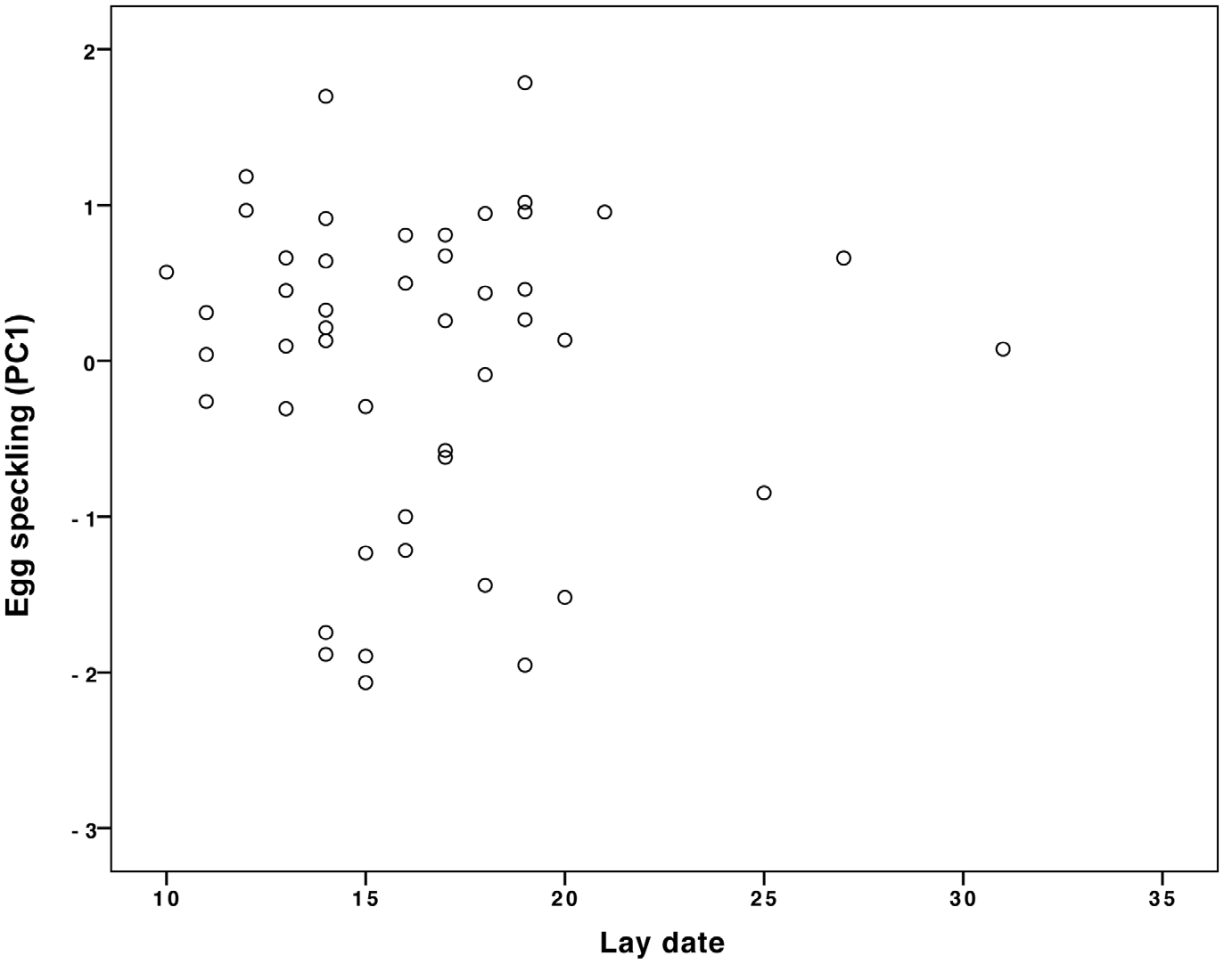
Female lay date had no effect on egg speckling (PC1). N = 47 (see text for statistics). Lay date is the date a female laid her first egg, shown here as the number of days after 31 March 2009.

**Table 5:** General Linear Model showing that heavier females laid less speckled eggs (N = 25 nests).

*Dependent – Egg speckling (PC1)*	B	df	F	95% L conf. int.	95% U conf. int.	P
Intercept	−0.03	1/23	0.02	−0.33	0.41	0.89
Female mass	−0.55	1/23	9.0	−0.90	−0.18	**0.006**
Female tarsus length	−0.32	1/22	2.35	−0.74	0.07	0.14
Male mass	−0.12	1/22	0.38	−0.50	0.25	0.54
Egg mass	0.04	1/22	0.04	−0.15	0.77	0.84
Lay date	0.04	1/22	0.04	−0.50	0.31	0.84
Clutch size	−0.04	1/22	0.03	−0.41	0.53	0.86
Male tarsus length	−0.02	1/22	0.01	−0.44	0.35	0.91

A third problem with tests of the SSEC hypothesis is that we still do not know whether there is enough light in the nest for birds to discriminate and evaluate egg speckling patterns, particularly for species that nest in holes and cavities where there is little light [7], [37], [38] and where females cover eggs during the laying period. This crucial assumption of the SSEC hypothesis remains questionable. Two recent studies explicitly tested this assumption in nests of blue tits [39] and spotless starlings *Sturnus unicolor* [40] and concluded that egg detectability depends entirely on numerical assumptions about light intensity in low light conditions. Consequently, both studies were inconclusive, demonstrating that a lack of physiological information in birds prevents us from determining whether egg color and pattern differences may be perceptible in dark nests. The studies emphasized increased “exploration of visual performance in the real conditions of the nest” [39] and cautioned that visual modeling alone “cannot, at present, be used to predict egg discrimination ability…under low light conditions” [40]. Some behavioral evidence suggests that bird parents are capable of responding to nuanced differences in nestling coloration even in the low light conditions of cavity nests [41], [42], but there is no clear consensus on whether the same may be true for subtle differences in egg appearance [43].

To address the main difficulties that have hindered previous tests of the SSEC hypothesis, here we combine rigorous experimental manipulation with objective assessment of egg patterning to determine whether protoporphyrin-based egg speckling is indeed a sexual signal to males. Great tits are an ideal study species for this test because males are expected to invest carefully in reproduction, and thus respond to signals of female or brood quality, since male care is extensive and obligate [44], [45]. Moreover, great tit females cover their eggs with nesting material during laying [3], so we know that the eggs cannot be seen by the male during this time. Once incubation occurs (and the eggs cross-fostered), the eggs are left uncovered while the female is off the nest ([46]; pers. obs.), so there is ample opportunity for the males to see the eggs before they hatch.

Whereas most studies testing the SSEC hypothesis for speckling have been correlational or at best have employed single cross-fostering, we performed cross-fostering at both the egg and hatchling stage to ensure that the effects of birth parent, incubating parent, and foster rearing parent could be completely separated. This double cross-fostering design ensured that paternal investment was not confounded by hatchling quality itself. In our experimental design, the egg patterns that the male bird observed and to which he could later respond did not belong to the chicks he later provisioned, guaranteeing that egg appearance and male investment were appropriately decoupled. We used natural eggs rather than artificial eggs to avoid the pitfalls associated with using painted models. Furthermore, we employed digital UV-sensitive photography specifically calibrated to bird vision to quantify egg patterns objectively, applying a recently developed image analysis technique to evaluate the 2D spatial arrangement of markings on the egg. Thus we were able to test whether male provisioning rates were correlated with the pattern displayed on the eggs incubated by his partner, independent of all other potential confounding variables. We did not perform visual modeling to determine how much birds can see in low-light nests since this has been performed comprehensively elsewhere [39], [40] and the results to date have been inconclusive. Instead, we designed an experiment to determine definitively whether or not great tit males respond to potentially perceivable differences in egg speckling.

## Materials and Methods

### Ethics Statement

This study complied with UK laws for animal research. Fieldwork was carried out under licenses from Natural England (20102731), the British Trust for Ornithology (C/4851), and the Home Office (PPL 80/2316).

### Cross-fostering Design and Video Analysis

In April and May 2009, experiments were conducted in Burnt Farm Plantation and the adjoining Short Nursery Plantation (lat.  = 52.23°, long.  = 0.04°), and Madingley Wood, Cambridge (lat.  = 52.22°, long.  = 0.05°) (Figure 1). Both are areas of mixed deciduous woodland, separated by approximately 1.6 km, covering a total area of 23 ha and containing 145 similar ‘woodcrete’ nest boxes (Figure 1). All nests were visited daily during egg-laying. The first four eggs of each clutch were photographed with a UV-sensitive camera specifically calibrated for bird vision (see details below). Although the degree of egg speckling can change throughout the clutch [47], we measured only the first four eggs 1) to ensure that we measured, for all clutches, the same number of eggs and eggs of the same lay-order, and 2) to minimize disruption at the nest. Clutch size in this population ranged from 6–10 (mean  = 8.1 +/− 0.19 SE). To confirm that the first four eggs were an appropriate representation of an entire clutch, we photographed five whole clutches upon clutch completion. Subsequent analysis showed no differences in the means of all four pattern variables (see details below) if calculated based on the first four eggs or based on all eggs in the clutch (paired t-tests; p > 0.05 for each pattern variable), so we can be confident that the first four eggs reflected the pattern characteristics of the entire clutch.

Eggs were photographed outside the nest box on a platform lined with black felt. To control for light conditions, all images included a Spectralon gray reflectance standard. Eggs were weighed using a digital balance (‘My Weigh Durascale’) every two days after the 5^th^ egg was laid, and the final mass achieved after clutch completion was used to determine average egg mass. At the start of incubation, when eggs were warm and uncovered, all eggs in a clutch were cross-fostered with another nest which began incubation on the same day and differed in clutch size by not more than two eggs. Note that great tits typically cover their eggs during the laying period but not during the incubation recess ([6]; pers obs), but see [39], so the male’s best opportunity for visually assessing the eggs is actually during the incubation period.

Upon hatching (day 0), each chick was individually marked by cutting one or two of the tracts of down on the head and back in a unique combination and then weighed with a digital balance to 0.01 degree accuracy. Chicks were weighed every two days between day 1 and 15. On day 1, nestlings were swapped between broods and a small blood sample was taken (<5 microliters). Cross-fostered chicks were of as similar a mass as possible and matched for mass rank, as in [48]. Each nest ultimately contained chicks from two different nests and none of its own. A total of 47 nests were included in the analysis on chick growth. Due to hatching failure and nest desertion, we do not have growth data for the original eggs that some (incubating or foster) females laid. Therefore the sample size for each stage of the experiment is fewer than the total number of nests involved. The final analysis included chicks from 42 original nests fostered into 42 nests for the incubation stage and fostered into 35 foster nests on day 1 for the entire provisioning period.

Videos were taken at the nest on days 9 (two hours in the afternoon) and 10 (two hours in the morning) of the chick-rearing stage. Nest box cameras were installed in the conical dome at the top of the nest box the day before filming began. Camera cables ran down to the ground through a notch in the side of the box front and were connected to a portable hard drive video recorder (Archos Pocket Video Recorder). Videos were analyzed using the computer program AVS Video ReMaker. The sexes of the parents were determined by crown and breast stripe coloration and confirmed via behavioral cues (*e.g.*, brooding and nest sanitation performed by the female). The average number of times each parent visited per hour was recorded. The length of all prey items was measured to the nearest millimeter with a 150 mm ruler, using the length of the bird’s beak on the screen as a standard: male beak length  = 13.31 +/− 0.44 SD, female beak length  = 13.36 +/− 0.50 SD, taken from [49]. Calibrating the beak dimensions in our videos against these published beak length estimates provided an approximate but not precise measure of prey length, as beak length in great tits is plastic and can even change within individuals over time [50]. Nonetheless, this approach is more accurate than ordinal scoring of prey items, *e.g.*, [51], [52], and – given that we were unable to measure beak length of all individuals in our population – provided a good baseline for prey size comparisons. We then calculated the average prey size delivered by the male and the average prey size delivered by the female. We calculated ‘prey delivered’ per hour by multiplying visit rate by average prey size.

Adults were caught using mist nets at each territory in March (N = 28) as part of another experiment that manipulated plumage color of the parents within the natural range. This treatment had no effect on any of the results reported here. We also caught adults at the nest on day 12 of chick rearing (N = 35) using a spring trap that was temporarily held in place between the door and the nest box. Body mass, tarsus length and wing length were measured in each case. We used the March body mass as a measure of quality prior to breeding, since mass on day 12 is affected by subsequent parental investment. Nevertheless, the results were qualitatively similar if we used female mass during chick rearing instead.

### Chick Sexing

A blood sample (<5 microliters) was taken from each chick on day 1. Molecular sex determination methodology was based on [53].

### Image Analysis and Pattern Quantification

We analyzed egg patterns following methods described by Stoddard and Stevens [35]. We used a Fujifilm IS Pro ultraviolet (UV)-sensitive digital camera with a quartz CoastalOpt UV lens (Coastal Optical Systems). The camera was fitted with a UV and infrared (IR) blocking filter for photographs in the human visible spectrum (Baader UV/IR Cut filter; transmitting between 400–700 nm) and with a UV pass filter (Baader U filter; transmitting between 300–400 nm) for UV images. We included a Spectralon gray reflectance standard (Labsphere, Congleton, UK) in all images. Photographs were taken at the same distance from the eggs to ensure that the markings were at the same scale. In birds, pattern and texture perception are thought to be a function of achromatic (luminance) vision, which is encoded by the double cones [54]. Therefore, we evaluated pattern in terms of this luminance channel, but we also examined UV images to make certain that we were not overlooking pattern information elsewhere in the bird visible spectrum. Using blue tit spectral sensitivity curves [55], we calculated images corresponding to the relative photon catches of a bird’s double cones (Figure 2) [56], [57].

For each image of an egg, we evaluated three sub-images of equal size in the upper, middle and base portions of the eggs, respectively. We applied fast Fourier transformation to obtain ‘granularity spectra’ [58], [35] for each egg section (Figure 2), and from the spectra we calculated marking size and pattern contrast. Marking size is a measure of the predominant spot size on the egg and is measured in terms of the filter size applied in the Fourier transform: smaller filter sizes correspond to larger markings and larger filter sizes correspond to smaller markings (Figure 2). Pattern contrast is a measure of pigment darkness: higher values indicate more contrasting markings against the light egg background. Additionally, we thresholded all egg images to calculate pattern coverage and pattern dispersion [35]. Pattern coverage is the proportion of the egg covered with pigmentation and pattern dispersion is the standard deviation of pattern coverage among the three egg regions, with high values indicating unevenly pigmented eggs. In sum, we calculated marking size, pattern contrast, pattern coverage and pattern dispersion for each egg. Measures of egg speckling were log transformed to comply with assumptions of normality.

This pattern quantification method is objective in the sense that egg patterns were evaluated in a mathematical and repeatable way based on bird luminance vision and Fourier transform analysis, which breaks down information into different spatial frequencies much like early-stage visual processing in many animals [59]. We cannot say definitively that patterns are processed this way by birds, but recent empirical work provides compelling evidence that pattern information (as measured using this method) is an important predictor of both egg rejection and egg recognition behavior [34], [60].

We performed Principal Components Analysis (PCA) on the four pattern variables for each egg (in this population, marking size: mean  = 11.95, SE  = 0.82; pattern contrast: mean  = 581.77, SE  = 15.32; pattern coverage: mean  = 0.19, SE  = 0.006; pattern dispersion: mean  = 0.10, SE  = 0.005; N = 184 eggs). PC1 and PC2 had eigenvalues greater than 1 and cumulatively explained 75% of the variation. Each pattern variable contributed approximately equally to PC1, which explained 49.0% of the variation. High values of PC1 indicated eggs with large, highly contrasting spots that covered a high proportion of the egg and were unevenly dispersed; PC1 therefore provided a good overall measure of egg speckling intensity. PC2 explained 26% of the variation and high values corresponded to eggs with small, low-contrasting spots that covered a moderate proportion of the egg and were unevenly dispersed. PC1 and PC2 were not exact opposites of each other; high values for both reflected uneven speckle dispersion. We performed all statistical analyses on PC1, PC2, and all four pattern variables independently. The results of these additional tests were qualitatively similar to those obtained using PC1. For simplicity, we refer only to PC1 in the results and conclusion.

### Statistical Analyses

The unit of statistical analysis was each clutch, rather than each egg, so we calculated average pattern measures for each clutch from the individual egg measurements. To investigate the effect of egg pattern on male provisioning rate, it was necessary to consider both the eggs a male’s partner laid as well as the eggs she incubated. We used General Linear Models (GLM) to determine whether egg speckling affected the rate of male provisioning. We used a General Linear Mixed Model (GLMM) to determine the effect of the experimental treatment on chick growth. A random factor of original nest nested within foster nest controlled for multiple chicks within a brood. There were three ways that egg speckling could be related to chick growth: to the speckling of the eggs from which the chicks hatched (“original egg speckling”), to the speckling of the eggs the foster parents originally laid (“egg speckling foster parent laid”), or to the speckling of the eggs the foster parents incubated (“egg speckling foster parent incubated”). Since male provisioning behavior in response to egg appearance may change over time [16], we examined the effect of egg speckling on chick mass for 3-, 7-, and 15-day-old nestlings. All values except principal component scores (which are already standardized) were standardized using Z-transformations. When evaluating male provisioning rate (Table 1) and chick mass (Tables 2, 3, 4), we controlled for female provisioning rate since maternal investment can affect both male feeding behavior and chick growth [29], [61].

## Results

Males did not use egg speckling to determine their chick provisioning levels (Table 1, Figure 3). Neither male visit rate nor the amount of prey delivered was related to the extent of speckling (PC1), neither on the eggs laid by his mate (Figure 3A) nor on the eggs that she incubated (*i.e.*, the eggs he saw, Figure 3B). We searched for evidence that the extent of parental investment was influenced by egg patterning in the longer term by examining chick mass at day 3, day 7 and day 15 (see Tables 2, 3, 4). There was no significant relationship between chick mass and the degree of egg speckling at any age (Day 3: GLM independent variable: chick mass, dependent variable: egg speckling (PC1) foster parent incubated: N = 287 chicks, 47 nests, B = −.0.01, F = 0.01, P = 0.93; Day 7: GLM independent variable: chick mass, dependent variable: egg speckling (PC1) foster parent incubated: N = 266 chicks, 47 nests, B = 0.06, F = 0.48, P = 0.49; Day 15: GLM independent variable: chick mass, dependent variable: egg speckling (PC1) foster parent incubated: N = 236 chicks, 47 nests, B = 0.11, F = 1.58, P = 0.22).

Although we found no evidence that the extent of egg speckling influenced male provisioning behavior, we did find that egg patterning was related to female quality because heavier females laid less speckled eggs (GLM independent variable: female mass, dependent variable: PC1: N = 28, B = −0.56, F = 10.1, P = 0.004, Figure 4). The extent of egg speckling (PC1) was not affected by lay date, clutch size, egg mass, male mass or tarsus length (Table 5, N = 25; sample size slightly reduced here by controlling for other variables). It is particularly important to note that egg speckling was unaffected by lay date. Were this not the case, one could argue that females laying at the same time were likely to be of the same quality and therefore likely to lay eggs with similar pigmentation patterns; as a consequence, our cross-fostering manipulation may not have provoked perceptible changes in egg speckling patterns. However, because we explicitly controlled for “female lay date” in our statistical models (see Tables 1 and 5) and found that lay date had no effect on either egg speckling (Table 5) or male provisioning (Table 1), we can be certain that female lay date did not influence egg speckling. Figure 5 demonstrates that there was no relationship between lay date and egg speckling (GLM: N = 47, B = 0.008, F = 0.007, P = 0.93, adjusted R^2^ = 0.02).

Heavy females, which laid less speckled eggs, also laid heavier eggs (GLM independent variables: female mass, dependent variable: average egg mass: N = 28, B = 0.05, F = 5.07, P = 0.033). Heavy eggs produced chicks that were heavier upon fledging (Table 4). Although heavy females laid both bigger eggs and less speckled eggs, there was no direct relationship between egg size and speckling (GLM independent variable: PC1, dependent variable: egg mass: N = 46, B = −0.02, F = 2.33, P = 0.13). Heavy females, or females that laid less speckled eggs, were not better at provisioning chicks (GLM independent variable: female visit rate, dependent variables: female mass, PC1, brood size, lay date: N = 20, female mass and PC1, P > 0.7).

## Discussion

Our experimental results allow us to unpick the complex relationship between female quality, the extent of egg patterning, and male provisioning behavior. We found no evidence to support the SSEC hypothesis: egg patterns had no effect on paternal feeding behavior. We did detect a relationship between egg speckling and female condition: heavier females laid eggs that were less extensively patterned. However, egg speckling did not advertise offspring quality because egg pattern attributes were unrelated to the subsequent growth rate of nestlings when fostered to other nests. Instead, egg size was a much better predictor of nestling condition (because larger eggs yielded offspring that grew faster, see Table 4). Egg size was unrelated to the extent of egg speckling. Most importantly, we found no evidence that males responded to egg speckling when deciding how frequently to provision the brood. In short, although the extent of egg patterning possibly indicated some aspects of maternal quality, it did not reveal nestling condition and males did not respond to these potential advertisements.

Why don’t males pay attention to egg patterns? There are several possibilities, all of which indicate that the quality of information encoded in egg patterns is likely to be very limited compared with other available cues. For a start, the nest environment could be too dark for the male to derive meaningful information from eggshells [39], [40], perhaps because light levels within a cavity or nest box fall to the mesopic range, where avian photoreceptor functionality is greatly reduced [7]. Upon entering a cavity, a bird may experience a 1000-fold reduction in light, which – even with adaptation to the new dim light level – may render detailed color and pattern discrimination impossible [7]. Therefore, the potential signaling capacity of speckles would be nullified by constraints on avian vision. Our results add to the growing body of work suggesting that nest cavities may really be too dark for birds to discern subtle differences in egg coloration and patterning [7], [36], [37], [62], but see [40]. Someone skeptical of this conclusion might argue that natural nest cavities (conditions under which signaling may have evolved) may have more light than man-made nest boxes (experimental conditions), so our use of nest boxes may have unfairly blinded males to information that is naturally present. However, nest boxes are not necessarily darker than natural cavities and indeed the reverse may be true: a comparison of nesting microclimates in a hardwood forest demonstrated that light was 25–209 lux higher in nest boxes than in natural cavities [63]. In our study, we did not perform visual modeling to determine how much birds can see in low-light nests since this has been done recently [39], [40] and the results have been inconclusive. In general, very little behavioral work has been done to test bird vision in low-light environments and it remains unclear whether physiological mechanisms other than color and luminance discrimination could be at work. Future research in this area will greatly help to clarify what predictions can realistically be made about signaling capacity in low-light nests.

A second possibility is that males may have very limited opportunities to obtain a clear view of eggs, even during the incubation period when eggs are left uncovered while females are away foraging, but see [39]. Instead, males may rely on other indicators of female quality that are easier to perceive, such as plumage or provisioning behavior, when regulating the extent of investment in the brood. Great tit parents certainly respond to one another’s behavior when negotiating parental care during chick provisioning [29], [61]. Males are likely exposed to multiple sources of information about female and offspring quality, both before and after hatching. Together with prior field experiments [29], [61], our results show, perhaps unsurprisingly, that male provisioning at the nest is most strongly influenced by current and salient signals from females and offspring rather than by out-of-date information subtly advertised on the egg [16].

If egg speckling does not signal female quality, then what is its function in hole-nesting passerines? Gosler and colleagues have proposed a structural role for protoporphyrin speckling [6], [47], suggesting that increased protoporphyrin deposition may compensate for reduced shell thickness caused by limited calcium availability. This may be particularly true for species, such as great tits, that lay eggs with pigments incorporated into the shell structure as opposed to superficially applied to the egg’s outermost layer. These integrated pigments appear to endow the shell with increased fracture toughness relative to non-pigmented areas of the shell [64], although the precise mechanism by which this occurs remains unclear. In non-passerines, support for the structural hypothesis comes from recent work on sparrowhawk *Accipiter nisus* [65] and northern lapwing *Vanellus vanellus* [66] eggs, which has shown a correlation between pigmentation and thinner areas of the shell; in sparrowhawks, shell thinning presumably resulted from calcium deficiency induced by DDT contamination. In contrast to these findings, the relationship between egg speckling and calcium availability is not always straightforward in great tits [67] or blue tits [68] and a structural role for pigmentation may not apply broadly to other taxonomic groups [69], [70]. Thus, the structural function hypothesis remains a viable alternative to signaling explanations for pigmentation, but more work is required to test its generality. In particular, it will be productive to incorporate objective techniques for quantifying pigmentation intensity and distribution and to determine how these measures correspond to protoporphyrin concentration [70].

Protoporhpyrin speckling might serve an antimicrobial function [71], although this is unlikely to explain egg pigmentation in cavity-nesting species because the process requires light activation. The cavity nest similarly rules out the possibility that egg patterning confers much protection from solar radiation [72] or predators [2]. Perhaps egg speckling is simply a by-product of female quality, a physiological artifact that does not convey much useful information. We observed a relationship between egg speckling and female mass, but egg speckling itself does not appear to affect a female’s reproductive quality, since it was not related to the mass of the chicks hatching from eggs she laid or reared (Tables 2, 3, 4). Alternatively, perhaps speckling is indicative of environmental conditions in the nest. A new hypothesis in great tits suggests that intraclutch variation in protoporphyrin patterning may reflect a female’s anemic condition, which changes over the course of egg-laying and is exacerbated by the presence of blood-sucking ectoparasites [73]. This recent discovery highlights the importance of understanding the mechanistic basis of pigment production, about which we still have much to learn [22], [36]. Especially in the case of pro-oxidant protoporphyrin, whether speckling reflects poor or good female health remains challenging to predict. We found that heavy females laid paler, less speckled eggs, lending some credence to the idea that high-quality females in good condition are better equipped to remove possibly harmful protoporphyrin molecules from the system [24], [25], thus laying paler, less speckled eggs. However, given how prevalent red-brown protoporphyrin is in avian eggs, both as a background color and in patterning, we find it unlikely that protoporphyrin is universally detrimental. Indeed, many birds rely heavily on protoporphyrin pigmentation to produce camouflaged eggs or eggs with elaborate signatures [1], [2]. Clearly, much more work must be done to evaluate the physiological, chemical and genetic mechanisms underlying protoporphyrin production and deposition.

Finally, it is possible that egg speckling in many small passerines was adaptive at some point in evolutionary history (perhaps for crypsis prior to cavity nesting) and has been retained even though it serves no obvious functional or adaptive role now and because it is not costly to produce [74]. In sum, the function of egg speckling in great tits and other hole-nesting passerines is not fully resolved. Given how much research attention has focused on the tremendous diversity of eggshell coloration and pattern in Aves, it is a marvel that this longstanding puzzle persists. Fortunately, new advances in visual modeling and digital imaging, as well as improved genetic and engineering techniques, make this an excellent time to evaluate a range of adaptive and non-adaptive explanations for the evolution of eggshell speckling.
